# Technological and Biological Systems’ Resilience: Observations and Learnings

**DOI:** 10.1007/s00267-025-02193-3

**Published:** 2025-06-02

**Authors:** Lindsay Robertson, Alan Bond

**Affiliations:** 1 Massey University, Palmerston North, New Zealand; 2 University of East Anglia, Norwich, UK

## Abstract

Technological systems have become progressively more complex, indispensable and ubiquitous, as has the inevitability of failures. These appreciations have generated increased interest in resilience. At present, the resilience of technological systems is highly dependent on ad-hoc and abstract problem solving provided by humans, and specifically their agency to repair damage: such dependence will inevitably become less practical as technological systems become more complex, and are impractical even now for systems that are inaccessible to humans. Biological systems, by contrast, typically demonstrate truly spectacular resilience, evidenced by the capability to self-repair deterioration and injury over millennia. Definitions of resilience commonly assemble multiple concepts describing the achievement of ‘resilience’ and the means by which this is achieved. Breaking down these concepts as they are applied to biological and technological systems allows useful analysis of the concepts that inhibit or promote resilience in technological systems. This paper attempts to learn from resilience processes as these are applied within biological systems, in order to clarify understanding of the basis for resilience of current and future technological systems. We propose that principles demonstrated to achieve high levels of resilience in biological system, can increase the resilience of technological systems.

## Introduction

### The Scope of Resilience to be Examined

This paper primarily considers the resilience of technological systems. The literature on resilience per se, is vast: a Scopus search on September 17th 2024 using the term “resilience” found more than 218,000 documents. A published literature review identified category clusters related to myriad topics, including environmental sciences and ecology, gerontology, psychiatry, finance, business and economics, engineering, neurosciences, education, amongst many others (Hosseini et al. 2016; Burn et al. 2016; Pooley and Cohen 2010; Connelly et al. 2017; Reid and Botterill 2013). Despite this extensive research effort, There is wide agreement that an adequate overarching definition of resilience does not yet exist, but nevertheless several definitions have been proposed (e.g., Aven 2010; Hollnagel 2022; Leveson 2020; Vogus and Sutcliffe 2007), that are dependent on the field of interest.

### Technological System Significance

The prediction (UN Department of Economic and Social Affairs 2018), that 75% of humanity will live in urban situations by 2050, indicates how important technological systems will become. This includes increasing dependence (for a large percentage of the global population) upon technological systems for daily necessities of life (food, water, power), for capacity to govern (financial systems, communications), for capacity to travel/commute (roads and fuel), for the capacity to regain health (drugs, and treatment), among many other current and future applications. This dependence upon technological systems, directly implies the severity of consequences that will follow if those systems cease to deliver their essential goods and services. Large numbers of examples could be quoted: Hardware vulnerabilities to major microprocessor types, Global Positioning System (GPS) functionality and energy supply vulnerabilities are typical.

### Resilience of Systems

For systems that have been stressed and have not failed, *ipso facto* these will return to their original format without intervention – and while this approaches an over-simplification, it also allows the clear identification of a specific aspect of “resilience” that both needs careful treatment and has not, as yet received adequate analysis: That is the response of a system to a subsystem failure.

### Significance of “Resilience”

Common definitions of resilience are always associated with “systems”, and commonly consider the capacity of complete systems to “bounce back” from a disturbance. It is obviously valuable to design subsystems to withstand progressively larger disturbances without damage, and if disturbances remain within tolerable limits, “resilience” becomes analogous to Hooke’s law and needs minimal further consideration. Cases where a disturbance exceeds tolerable limits do however need consideration.

Much literature exists on the topic of “risk” (e.g., Aven 2010), and primarily categorises combinations of the severity of a failure of a particular subsystem and the probability that such failure will occur (*i.e*., a disturbance exceeds the tolerable limit (Strigini, 2012). Without questioning the value of such an approach, we note that over a long enough time-frame, the probability of subsystem failure approaches 1.0, *i.e*., failure is inevitable!

If a system is dependent on a single subsystem, and that subsystem is “disrupted” to the extent it totally fails, then the (dependent) system must by definition also totally fail. Even when a system’s initial design eliminates the possibility of immediate cascading failure (E_1_ > 0) (Robertson et al. 2017), an inevitability still exists: wherever a system depends upon a plurality of subsystems, the exposure of the parent system increases as each subsystem fails, and inevitably approaches the state where a further subsystem failure will cascade upwards to system failure.

A conceptually clear conclusion emerges: unless a mechanism for the recreation or repair of a failed sub-systems is extant, any “system” will inevitably fail. It is proposed that this is a conceptually clear aspect of “resilience” that needs consideration. It is proposed that recognition of these two issues, *viz* the significance of technological system failure, and the inevitability of subsystem failure, have collectively been foundational to the current interest in “resilience”.

### Resilience Observed

Within a biological system such as a human body, sub-system failures such as those caused by a minor cut, are very common. A minor cut does not result in death by exsanguination, and if human society is considered as a “system”, it is reasonable to note that despite the death of individuals, such a “system” has survived for tens of millennia.

By contrast, there are few technological systems that survive more than a small number of decades.

We must not conclude that biological systems have unlimited resilience: there are clear bounds to this resilience but nevertheless their demonstrable resilience is orders of magnitude higher than for current technological systems.

### Resilience Described

As a seminal description of resilience, Aven (2010) states that “… resilience is defined as the ability of the system to withstand a major disruption within acceptable degradation parameters and to recover within an acceptable time…”: This typical description is not disputed but the statement can be usefully dissected:

- If the nature/scale of “disruption” were within tolerable limits of the subsystem to which it was applied, then (as for the spring stressed below elastic limits) removal of the disruption will immediately (“within acceptable time”) demonstrate no residual degradation at all. Efforts to expand the magnitude of tolerable disturbance (increase robustness) are laudable, but for consideration of resilience, it is reasonable to focus attention on disruptions that cause permanent sub-system change!
- The concept of “recover” carries an implicit assumption that the “system” contains both the problem-solving capability, access to materials, and the agency (manipulative capability) to effect the repair/replacement of the “disrupted” subsystem (publications that consider the term “resilience” but also Hollnagel (2022), Leveson (2020), Vogus and Sutcliffe (2007) and Yu et al. (2020) actually demonstrate that the resilience espoused in those publications has its basis in the abstract problem solving capability plus the manual dexterity (agency) of human beings): We note that the Voyager spacecraft carries no workshop, has no on-board technician nor spare-part inventory, and so damage to its main antenna (which would be simple for a technician on earth to repair) cannot be repaired and certainly not “within an acceptable time”.
- The concept of “within an acceptable time” also contains two concepts. Firstly, the end-user’s perception of the time-frame within which the system’s failure does not have consequences for them, and secondly, the consideration of the time-frame within which additional sub-system failures are likely!

Such disaggregation of underlying concepts allows useful analysis of the real capability for resilience.

### Paper Scope and Aims

Noting the significance of technological systems and the effect of subsystem failure upon a dependent system, and additionally noting the inevitability of subsystem failure, we have assessed that it is useful to consider the response of technological systems to subsystem failure as a distinct field.

The specific categories of capability (Vogus and Sutcliffe 2007) implicitly assume that the target systems are, in fact, socio-technical systems. We might consider a large power station, designed with safety systems, and complete with operations and maintenance staff, spares inventory, and maintenance planning as such a socio-technological system. Such systems may show a capacity to recover from disruptions (minor sub-system deterioration and or component failure) but they have also become increasingly dependent on highly skilled operators and global supply-chains. For many other essential systems, human intervention is becoming increasingly impractical.

Based on these observations, the aim of this paper is to consider how long-term resilience of technological (but not necessarily socio-technological) systems might be improved by systematically disaggregating and codifying the concepts that are found to occur effectively and semi-automatically, within biological systems. Our research achieves this by a process of conceptualization of the functional components that actually produce the outputs that are commonly ascribed to ‘resilience’ for biological systems.

The next section briefly sets out the methodological approach, before analysing resilience concepts within biological systems, followed by an analysis of technological resilience against similar concepts. We conclude with consideration of possible strategies for delivering improvements in technological system resilience and a concluding statement regarding the significance of resilience.

## Methods

### Common Resilience Concepts

While examples will be mentioned, this research is not based upon case-studies but rather attempts to identify principles that can be applied across cases. Concepts and definitions of resilience are widely published and provide the foundations for this work. This study considers some of those basic elements that underlie the concept of resilience and that differentiate it from the study of risk and from the study of robustness. It seeks to compare the approaches and features of biological and technological systems and from that to highlight issues that could improve the resilience of technological systems. Given the vast (and often conflicting) literature on resilience, a systematic literature review is both impractical and potentially unhelpful. Instead, the authors draw on many years of conducting research into resilience concepts, aiming to synthesise that learning to achieve the aims of this paper.

### Analysis of Resilience in Biological Systems

We note that biological systems have routinely demonstrated their spectacular resilience in the absence of either human-level abstraction or specific agency (dexterity).

This paper will conceptualise the mechanical aspects of a biological organism’s self-repair and self-replacement capabilities by distilling a set of components underpinning the resilience of those capabilities. That is, identifying those capabilities that are foundational to resilience in technological (or socio-technical) systems. We will effectively consider biological systems as highly advanced technological systems, and will examine the mechanisms for survival, functional preservation and damage repair. We note that while the mechanisms by which biological systems achieve resilience have been studied extensively (Baserga 1985), and at a greater depth than can be traversed in this paper, many issues remain unclear; for example, why can a person not re-grow a limb to replace an amputation (Elchaninov et al. 2021).

To conduct the conceptualisation, we followed the approach set out by Jabareen (2009): 1. identifying aspects of resilience typical of biological systems; 2. deconstructing and categorising the aspects into themes; 3. integrating themes; and 4. synthesis, re-synthesis, and making it all make sense.

In this way, the knowledge on biological system resilience is deconstructed and then synthesized into themes that can be used to further consider the extent to which mechanisms used in biological systems can (currently or in the future) be applicable to technological systems.

### Analysis of Resilience in Technological Systems

Analysis will make use of basic benchmarking to compare technological systems to biological systems, with resilience themes drawn from analysis of biological systems acting as the benchmark. The use of benchmarking as a means of comparative analysis is widespread in the scientific literature in many fields, for example, corporate credit rating (e.g., Huang et al. 2004), waste management (e.g., Wilson et al. 2012), medicine (e.g., Ziegenhain et al. 2017) and transport (e.g., Debnath et al. 2014).

Comparison of the resilience of technological and biological systems, is constrained by the following questions:

- What evidence suggests that the lessons from biological systems are optimal across a wide range of situations (noting that it has emerged within a specific environment that may not be replicable)?
- What evidence suggests that the lessons from biological systems can be transferred to technological systems? If there are fundamental issues that prohibit transfer, then is the comparison not useful?
- What evidence suggests that the lessons from biological systems are optimal for transfer to technological systems? Is there any reason to assume that there could not be other options that may be better than a translation of biological system approaches?

Finally, this paper will examine the extent to which current practice and also current trends in the design of technological systems either converge towards-, or diverge from- the themes that characterize resilience in biological systems.

## Embedded Resilience Concepts

In order to establish a clear line of logical argument leading to a conclusion, it is useful to clarify some concepts that are either commonly assumed, or aggregated with others.

### Defining Systems and Sub-systems

The significance of technological systems has been noted earlier: it is now important to consider the concept of a “system” which is identical to the concept of a “sub-system”. Although sometimes tedious, it is possible to unequivocally define a subsystem using the concept of a system boundary or bubble (Haskins and Fet 2023; Backlund 2000) being a nominally-thick enclosure that allows definition of all flows of information and material into and out-from the enclosed processing functionality. Although an animal’s skin, and a cell-wall have more complex functions, these have analogies to the system boundary concept.

### Defining “Failure”

It is also possible to define the concept of subsystem failure. A subsystem normally operates without hysteresis - external inputs (pressure, temperature, etc.) will fluctuate within nominal ranges and output expectations will rise and fall, causing input expectations to rise/fall based on some continuous transfer function (Van Den Hof and Schrama 1993). “Normal operation” is however clearly achieved when a return of external conditions to some pre-disturbance level causes the subsystem operation to return to its pre-disturbance level. For any time-frame following a subsystem failure, the converse pertains - regardless of whether external conditions return to some pre-disturbance level, system operation never returns to “normal”: infinite hysteresis (Morris 2011) has been acquired and mathematically, the transfer function is non-continuous across some defined time-frame. A clear definition of “failure” is achieved.

Despite all of the best (and laudable) current and future efforts to increase the robustness of any system (biological, ecological or technological), the most basic laws of engineering (second law of thermodynamics) shows that entropy always increases. The inevitability of subsystem failure is therefore proven and only avoided by input from a larger system (whose entropy will be raised). The foundational nature of the issue must not be underestimated.

### Avoidance of Issues that Preclude Resilience

There are at least a small number of issues that simply preclude a resilient response from any technological system:

- If a system is completely dependent on outputs from one subsystem, then by definition a failure of the single subsystem will cause the system to fail to produce its output.
- Even where a system is dependent on multiple subsystems, if all of those subsystems are dependent on a single environmental condition or input, the failure of the condition/input will cause the system to fail.

The above issues can be quantified in terms of system “exposure” (Robertson et al. 2017) and are closely associated with concepts of survivability. In addition to the above, further issues that preclude resilience include:

- The lack (loss) of critical material. If a manned spacecraft loses all stored oxygen to space, options for resilient response are lost, for all practical purposes.
- The lack of agency (combining the concepts of situation awareness (of a fault, and associated drive to rectify), abstract problem-solving and of manipulative capacity). Without these, a disruption (failure) will simply remain extant indefinitely.

All (technological, biological and ecological) subsystems provide inputs to dependent systems: In any case where a system’s performance is dependent on inputs from a subsystem, then we can *ipso-facto* conclude that a subsystem failure (as defined above) will cause the failure of the dependent systems. The logical extension of this concept leads to the evaluation of a system’s “Exposure” (as per Robertson et al. 2017). The possibility of cascading failure is therefore linked to system configuration, and so it is at least conceptually possible to develop system designs that reduce exposure and eliminate the possibility of cascading failure. This conceptual possibility does not consider either economic or practical desirability, but must be noted as a theoretical boundary to a problem. The study of system “exposure” has shown the inevitability of cascading failure where a system is dependent upon a subsystem. Cases where multiple subsystems are loaded to the level where a single subsystem failure inevitably loads other subsystems beyond their tolerable limit will also inevitably cause a cascading failure (e.g., Vaiman et al. (2011), relating to the collapse of the green bank radio telescope). Much has been written about supply chain resilience (Novak et al. 2021; Wieland and Durach 2021) based on the optimisation of stockpiles held at locations within a supply chain. It can be observed that no stockpile is infinite and so if the means of replenishing the stockpile ceases to operate, the stockpile will inevitably be depleted. A stockpile is useful, but without true resilience it is always a limited solution. It is common for high-criticality technological systems to incorporate a backup sub-system, i.e., a duplicated sub-system which is not normally in service but can take over the duties of the operational subsystem upon the latter’s failure (Chen and Crilly 2014). High criticality systems may justify the costs of such design redundancy but for most systems the cost (capital and maintenance) is unacceptable even for double or triple redundancy and becomes prohibitive beyond those limits. It is important to review the basis for design redundancy: for an aircraft in flight, the availability of a redundant control system may allow the flight to land safely at a facility where the main control system can be repaired. For the Voyager space-craft, once the main system fails, the backup continues but once the backup fails the whole craft fails: the backup is effectively a system life-extension capability. Both stockpile and design redundancy approaches are temporary solutions that are useful if, and only if, they gain enough operational time to allow another mechanism to effect repairs before some cascading effect (Teixeira et al. 2020): otherwise these are no more than expensive solutions that achieve limited system life-extension. We conclude that it is important to elucidate the factors that preclude resilience. These may be configurations in which systems are unconditionally dependent on specific subsystems (that can fail) or environmental specifications that could preclude operation of systems and all subsystems (such are labelled as “existential threats” and define outer limits within which resilient responses are conceptually possible).

### Pre-requisites for Resilience

The previous section has noted the significance of ensuring that a subsystem failure (which is inevitable, eventually) does not automatically result in system failure: we can however also note that unless a subsystem failure is remedied the progression towards system failure is actually inevitable. This is again an observation that has a well-founded theoretical base.

The concept of subsystem failure has been explored (and is simple to define): recovering the original functionality therefore requires the replacement/recreation of the failed subsystem. In a subset of cases, the raw material from the failed subsystem may be able to be re-worked for this purpose. In all cases, however, replacement/recreation has a necessary condition, *viz* the availability of the material and knowledge required. If these are unavailable/absent, the failed subsystem will quite simply remain perpetually in its failed state.

The concept of “availability” requires careful definition: Photosynthesis requires atmospheric CO_2_ and photons that are both almost unconditionally available - but considerable effort would be required to find specific semiconductor devices, and critical masses of fissionable material are hopefully very difficult to acquire.

### Sufficiency for Resilience (Issues that Ensure that Resilience Happens)

The capacity to detect failure, initiate and execute measures in response to a failure provide the “sufficiency” for resilience and these capabilities are proposed to be separable from the factors that preclude resilience and are also separable from the basic capacities that are necessary for resilient response.

It has been already noted that publications related to technological resilience commonly assume (implicitly) that the basis for achieving resilience is the application of abstract problem solving plus the dexterity (agency) of human beings (Brown and Westaway 2011; Romero and Stahre 2021). Resilience of systems involving both technological systems and human beings should be categorised as “socio-technical systems”. We propose that the capabilities upon which resilience is based, can be further subdivided into “awareness of desirability”, “capability for situational awareness”, “capability to pose novel options”, and “capability to evaluate performance of hypothetical solutions (thought-experiments) - followed by capability (dexterity/agency) to implement the hypothetical solution that has emerged.

### Conclusion Re-categorization

In summary (categorizing themes), we note that if a subsystem failure inevitably cascades to cause the failure of the parent system, resilient response is highly unlikely. Assuming that a subsystem failure does not inevitably cascade, the necessities for a resilient response may- or may-not be accessible. Assuming that a subsystem failure does not inevitably cascade, and further assuming that the necessities for a resilient response are available, we propose that the abstract awareness of failure, the need for repair and capability to manipulate material supply the sufficiency to achieve a resilient response.

### Static and Dynamic Environments

All the concepts considered in turn above have considered resilience within the assumption that the external environment has remained basically static (notwithstanding that a disturbance may have caused a subsystem failure). A consideration of resilience within an environment that is not static, is a very significantly different and larger topic that will only be treated superficially within this paper.

## Resilience in Biological and Technological Systems

### Biological Systems’ Resilience

The introduction to this paper noted that whereas biological systems have demonstrated capability to survive over millennia, technological system survival has seldom exceeded decades. The observation suggests that there is significant value in understanding how and why biological systems have achieved their spectacular results.

It is valuable to elucidate issues that might be taken for granted in the context of a biological system, and it is also valuable to use the same terms as would be applied in other circumstances (e.g., technological systems).

As previously noted, without a capacity for replication, common small injuries would inevitably accumulate (and an organic system would see a progressive and inevitable decrease in capacity). With an average cell life of 7–10 years (Sender et al. 2021) the capacity for replication of biological subsystems at this most basic level is absolutely essential: No individual could survive if the majority of their cells died within 7–10 years, and no human society could survive if individuals’ lifetimes were limited to 7–10 years.

Biological systems in the context of this paper are exemplified by human society. In order to illustrate the points made, the following diagram traces one subsystem. The circulatory system, the immune system, the reproductive system each have similar layered capabilities to replicate failed subsystems.

It is useful to consider a cell as a subsystem; we may then consider: how the necessity for replication is identified and signalled; what are the pre-requisites for replication; how the actuality of replication is initiated; how the totality of replication is effected; and, how replication is regulated (controlled)?

**Fig. 1:**
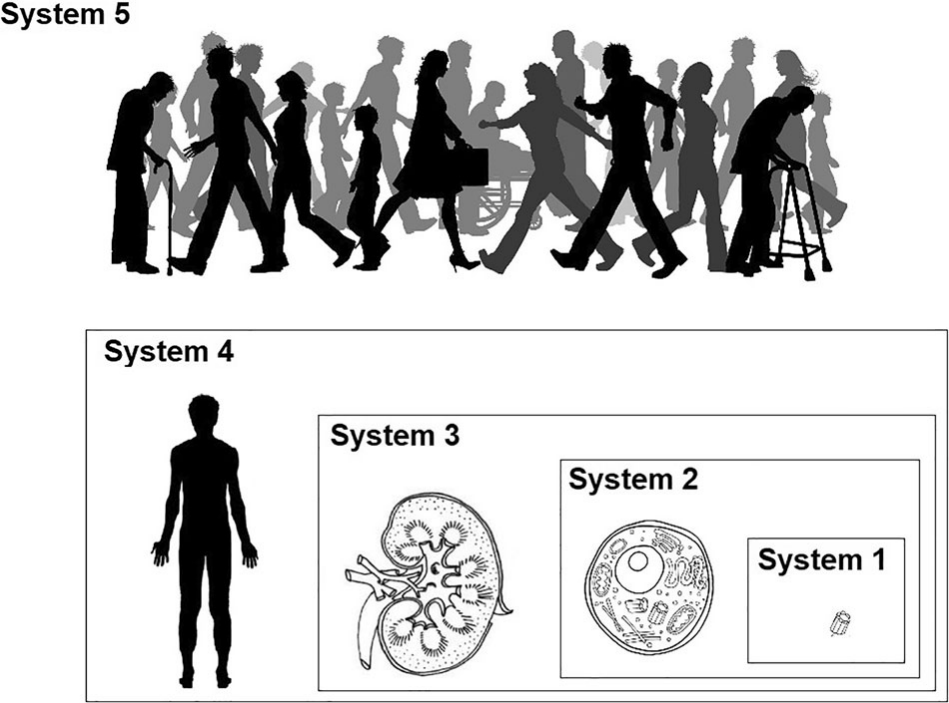
Layers of systems, typical of biological systems

Figure 1 illustrates the essential layered functionality of these system/subsystem groupings. This illustration (considering one typical organ) is a gross simplification of immensely complex processes, but is proposed as typical.

Considering the concept of resilience, we may note the essential distinctions between the layers: System 1 - Organelle: No capacity to replicate itself, and is a Single Point of Failure (SPOF) for a cell. System 2 - Cell: Is able to replicate itself. System 3 - Body Organ: No capacity to replicate itself, is a SPOF (with limited exceptions including kidneys) for an individual. System 4 - Organism: Is able to replicate itself. System 5 - Society: No capacity to replicate itself.

In summary, each layer that is not able to replicate itself, has a distinct layer above that is capable of replacing failed systems: Cascading failure options are strictly limited.

The following subsections reflect the different themes emerging from the analysis and the literature, as a means of benchmarking biological system resilience.

#### Avoidance of Issues that Preclude Resilience in Biological Systems

This arrangement also allows a consideration of “survivability” (Smaili et al. 2008; Knight et al. 2023): An organ is unlikely to fail because of the failure of a single cell, and a society is unlikely to fail because of the failure (death) of a single individual.

Most organs within an organism (e.g., the human body) have billions of cells (Sender et al. 2021): The functionality of the organ, of which the cell is a part, is achieved by the combined outputs of billions of cells. Because of this, the demise of even a large number of cells has minimal effect upon an organ (unless a common point of failure is affected).

It is significant to note that biological organisms do not use the “backup system” approach at all - organisms do not contain sub-systems that are functional but unused, pending failure of a “main system”. Biological systems rely totally on the multiple shared-load approach. There are multiple systems that each have (similar) layers.

#### The Capacity to Achieve Resilience in Biological Systems

**Fig. 2:**
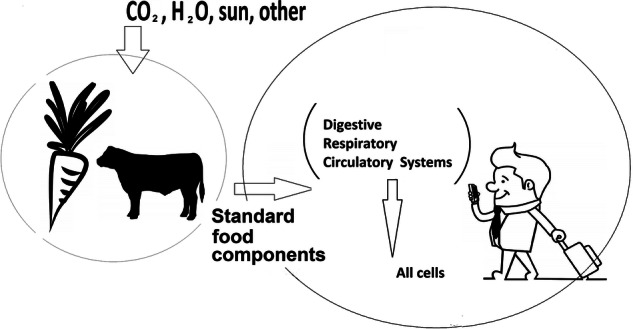
Availability of necessities for resilience

Considering Fig. 2: We may note that all of the resources needed to enable a cell to replicate are supplied via the organism’s bloodstream. This single, essential and ubiquitous resource-stream is in turn created within the organism. At each of these scale-points (levels), local processing including replication is possible using only basic resources that are almost unconditionally available. It may also be noted that each cell contains the full information needed to replicate the parent organism (DNA and chromosomes in each cell represent the complete DNA for the body - a DNA sample from either hair, blood, muscle will all uniquely identify the parent organism). This “design knowledge” is very widely disseminated, there is no case where a cell contains only the information needed for its own functioning or replication. Of equal significance (when considering a translation to technological systems) is the observation that an organ will typically contain very large numbers of essentially identical cells that all contain high levels of DNA commonality.

Noting the level of duplication of DNA across widely different species (de Crécy-Lagard and Hanson 2013) we may speculate that a large portion of the DNA is associated with functionalities that are common across species (cell survival/functionality, and cell replication). The DNA/Chromosomes of each cell also contain the capability (agency) that allow the cell to replicate itself using only the raw materials provided by the bloodstream. It can be observed that each type of cell within an organism (for example, liver, kidney and brain cells within a human) can each acquire all the needs for replication, from the same blood supply. and requiring only input of oxygen, water and basic food (the human body is extraordinarily adept at converting even the most basic food supply to generate all that is needed by the body’s cells. It may also be observed that basic digestible carbohydrates are produced by plants (capable of cell and organism replication), requiring only inputs of photons, water, CO_2_ and trace elements). The inputs that allow (are necessary for) resilience are standardised for all subsystems. The functioning of the DNA and chromosomes has resulted in a large body of literature, whose scope is beyond that of this paper. Two cogent points may be noted: a) At the lowest level at which replication/recreation can occur, the complete information-set that is capable of constructing the organism (assuming the unconditionally available streams), is located within every cell, and is carefully replicated every time the cell replicates itself. b) At the lowest level at which replication/recreation can occur, the inherent capability to replicate the specific cell in which the total information is held, is available. Both the capability exists, and also the constraint to only replicate the current cell. The ability to replicate a human-being, needs no proof, although the science of reproductive medicine continues to discover yet further wonders. The question of why most organisms seem to be unable to re-grow an amputated limb, is of significant interest to medicine (Kulebyakin et al. 2020) but we equally note that cancer is related to uncontrolled cell-growth. The significance of replicating the design-basis of the complete organism, within each cell, is immense: One might ask why an organism might not have evolved a more parsimonious approach to information storage (e.g., by replicating only the design information needed to replicate the particular cell, within that cell). This misses the key point, that the cell’s capability to survive and replicate is its primary goal (hence the large levels of commonality of DNA across species. This is in stark contrast to current technological systems, where the capability to replicate is either non-existent or vestigial.

#### Sufficiency for Resilience in Biological Systems

In the case where a cell dies from some unanticipated event, products of the cell’s death may contribute to triggering replication of other cells, but will also trigger “first aid” measures (e.g., blood clotting, immune system responses that prevent additional and cascading damage. There are also clearly cases where replication is not triggered by the demise of a cell, but by a signal that such demise is anticipated. It is quite clear that cell replication is not dependent on abstract problem-solving: regardless of how the replication is triggered and is executed, very accurate replication (without mutation) is a process that does not require ad-hoc problem solving within the duration of the replication process. Importantly, the need for ad-hoc problem solving during the process of replication has been avoided by a highly automated process. Regulation of replication is a topic of immense scientific interest, noting that the general term “cancer” identifies cases where replication is inadequately regulated.

#### Biological Resilience in a Dynamic Environment

The preceding analysis with respect to biological sub-systems, has assumed a relatively static environment. For situations where the environment is not static, it would be possible to assume a highly conscious process of imagineering, conceptualisation, thought-experimentation etc., ultimately leading to a revised solution, this is NOT the only possible approach. The use of sexual reproduction creates very large numbers of “copies” that are not identical, and relying upon an implicit assumption that some may be found to be better-able to survive/reproduce than others. This is the basis of Darwinian evolution and is effective where a marginally different environment either exists or emerges slowly, allowing a viable mutation to show a breeding advantage.

### Technological Systems’ Resilience

#### Avoidance of Issues that Preclude Resilience in Technological Systems

There are at least a small number of issues that simply preclude a resilient response from any technological system: if a system is completely dependent on outputs from one subsystem then, by definition, a failure of the single subsystem will cause the system to fail to produce its output. Even where a system is dependent on multiple subsystems, if all those subsystems are dependent on a single environmental condition or input, the failure of the condition/input will cause system failure. The above issues can be quantified in terms of system “exposure” (Robertson et al. 2017) and are closely associated with concepts of survivability.

Cases where multiple subsystems are loaded to the level where a single subsystem failure inevitably loads other subsystems beyond their tolerable limit will also inevitably cause a cascading failure (Vaiman et al. 2011).

Much has been written about supply chain resilience (e.g., by Novak et al. 2021): much of this relates to the optimisation of stockpiles held at locations within a supply chain. It can be observed that no stockpile is infinite and so if the means of replenishing the stockpile ceases to operate, the stockpile will inevitably be depleted. A stockpile is useful, but without true resilience it is always a limited solution.

The study of system “exposure” has shown the inevitability of cascading failure where a system is dependent upon a subsystem.

It is common for high-criticality technological systems to incorporate a backup sub-system, i.e., a duplicated sub-system which is not normally in service but can take over the duties of the operational subsystem upon the latter’s failure (Chen and Crilly 2014). The “cost” of creation of a technological subsystem is significant: there is a very well-established approach to scaling the capital cost of a subsystem, related to the capacity of the sub-system: This is a scaling-factor (^0.66), and the implications of the (simple) approach are profound: high criticality systems may justify the costs of such design redundancy but for most systems the cost (capital and maintenance) is unacceptable even for double or triple redundancy and becomes prohibitive beyond those limits. It is important to review the basis for design redundancy: for an aircraft in flight, the availability of a redundant control system may allow the flight to land safely at a facility where the main control system can be repaired. For the Voyager spacecraft, once the main system fails, the backup continues but once the backup fails the whole craft fails: the backup is effectively a system life-extension capability. Both stockpile and design redundancy approaches are temporary solutions that are useful if- and only-if they gain enough operational time to allow another mechanism to effect repairs before some cascading effect (Teixeira et al. 2020) : otherwise these are no more than expensive solutions that achieve limited system life-extension.

While it is not within the scope of this paper to survey current technological systems, there are numerous examples to suggest that systems with high exposure and low resilience exist, e.g. as noted by Bueger et al. (2022) and O’Reilly et al. (2018).

#### The Capacity to Achieve Resilience in Technological Systems

For many simple technological systems (consider an atlatl as an example) human capability to replace failed subsystems (the tool in this example) is made feasible because the natural resources for replacement are ubiquitously available, and the person’s skill and knowledge are the only additional requirements to effect the replacement. If the failed subsystem is simple farm equipment, a village-scale workshop may be able to achieve a replacement using bar-stock and common workshop tools. As the complexity of the subsystem increases, we inevitably come to a situation where a global-scale system is required to replace a failed system (either key components are only available at international scale, or the capabilities for manufacture of all subsystems is only available in specific parts of the globe and cannot be replicated close to the failure location).

The necessities for resilience (a consideration applicable to both biological and technological systems), can therefore be compared by considering the scale at which all essential (material, knowledge, agency) inputs can be practically made available to effectively replace a failed subsystem. For biological systems, highly localised knowledge-based (replicated DNA) and highly localised manipulation (expression) capabilities, make resilience (replacement of damage) possible via a general-purpose resource (blood supply) that is available to all cells. Similarly, the nutrients and other requirements are themselves generated locally (plant and animal sources) from equally basic and ubiquitous resources. By contrast, replacement of a local component of a technological system may effectively require a financial, transport, offshore manufacturing, offshore knowledge-base, and skilled labour resource - and hence only be practical at an international scale. This is an essential difference between resilience capabilities of biological and current technological systems.

#### Sufficiency for Resilience in Technological Systems

Even if a subsystem failure does not cascade, and even should replacement material exist, any failed subsystem will certainly remain in its “failed” state unless some action is taken to replace it, i.e., unless the “sufficient” condition for resilience is met.

It is currently common to find technological systems that, for example, automatically route communications past inoperative communication nodes (assuming that alternative routes exist): it is also common to find systems (such as supply-chain applications) that exploit stockpiles (until they are exhausted) if an intermediate supplier fails.

It is also common to find high-value systems incorporating diagnostic features that signal incipient problems.

It is still uncommon to find automated systems that act to replace the ailing subsystem - except where this action (sufficiency) is provided directly by human intervention. As previously noted, as technological systems become larger and more integrated, so the validity of dependence upon human capabilities becomes progressively more problematic. The emergence of Artificial Intelligence and additive/subtractive manufacturing systems suggest that these capabilities may be possible in the foreseeable future - but they are certainly uncommon at present.

For the vast majority of current cases, the “sufficiency” for resilience in technological systems is provided by human capacities for abstraction, and cooperative human agency for execution.

#### Technological System Resilience in a Changing Environment

Where highly specialized human knowledge is available, then basic human abstraction capabilities and cooperative agency may be used to adapt/re-purpose technological components and this capability (not present within biological systems’ capabilities) to achieve resilience. Similarly, human abstraction and cooperative agency may be used to recreate failed subsystems. Such capabilities become less practical as technological systems become larger, faster and more centralised, since the necessary knowledge becomes less common: this trend (expounded below) represents a divergence from the principles that enable resilience in biological systems.

#### Trends in Technological System Resilience

For many technological systems, economies of scale are available, providing competitive pressure towards progressively larger (and hence fewer) systems. Although it is a gross generalisation, the cost of a technological system is commonly scalable, as noted by, for example, Gerrard (2000), Böhm et al. (2020); neither material costs nor manufacturing costs are linear and therefore there is a strong incentive towards fewer systems of larger capacity.

Economies of scale therefore develop pressure to move from multiple smaller systems that would offer increased resilience, generate increased difficulty to replace a failed system and increase the vulnerability of a larger number of consumers.

For some technological systems there are currently minimum capital expenditure (capex) levels - for example an undersea fibre-optic cable.

Except for high criticality fields, the capital and operational cost of maintaining a backup plant (only used when the main plant fails) is excessive. As scale increases, the cost of a ‘standby system’ obviously rises and will ultimately become prohibitive. This is diametrically opposite to the biological systems’ approach.

Larger and fewer technological systems mean higher “exposure”, for users. The cost, to the technological system’s owner, may however be low (leading to “asymmetrical consequences’); if so, the owner may tolerate the existence of a high-exposure system if only it has a low outage rate (high reliability) and may not feel pressure to provide for the situation where a system fails.

Most large technological systems today are operated by commercial entities, and for many such there is commercial incentive to capture as much of the value chain as possible. Resilience would be enabled if alternative suppliers could provide capacity at multiple stages in the value chain but this would require standardisation of inputs, and would decrease the proportion of the value chain available to the commercial entity and so this option is resisted. For a consumer (whether an end-user or a system requiring inputs), vulnerability is reduced if multiple suppliers are able to offer the same goods/services. The consumer may benefit from the case where a single commercial entity develops an offering that cannot be matched by others - but the consumer automatically becomes vulnerable to failure of that single entity. Efforts to capture market share, and to capture more of a value-chain, are therefore significant factors that may therefore preclude resilience by increasing the “exposure” of technological systems.

Beyond simple artifacts, some layered standardisation has been achieved (shipping containers, rolled-steel sections, bolts, ASCII codes (current web standards) and ISO/national standards - but beyond these, the levels of standardisation are not only limited but under pressure from commercial drivers. Without standardisation of layers, the necessities for replication of failed subsystems (and hence true resilience) may only become available at national or global levels and from a limited numbers of suppliers: this is a trend opposite from the resilience pathway found in biological systems and does not promote resilience.

Trends in technological resilience are not static: technologies such as small-scale additive and subtractive manufacturing, Field-Programmable Gate Arrays (FPGA’s) as described by Fong et al. (2003) and others are examples of cases where capabilities that were once only available at national level, become available at much smaller scales and this can be seen as a trend (Robertson 2020).

## Discussion and Conclusions

### The Distinctive Needs for Resilience in Technological Systems

We have noted that subsystems always operate with varying inputs (including subsystem status). Over a finite range of inputs, subsystem responses can be described by a continuous function and within these “tolerable limits”, “bouncing back” will occur automatically and without any specific action. Even one single exceedance of a tolerable disturbance level will however cause a subsystem to be damaged/destroyed, without resumption of functionality should the disturbance be removed. In this failed subsystem case, “bouncing back” will never occur without specific actions. Noting that over a long time interval, a single exceedance of tolerable disturbance is inevitable, we have clarified a need to consider the resilience of a system under conditions of subsystem failure. Backup systems and intermediate product stockpiles may allow a system to continue to operate temporarily when a subsystem fails – but without a means of recreating the failed subsystem, stockpiles or other buffers will inevitably be exhausted and backup systems will also (sooner or later) fail. The failure of all subsystems will inevitably cause the failure of a dependent system – a recursive observation.

Humanity has developed a critical dependence on technological systems, and so the consideration of resilience as described above, is a significant issue.

### Comparisons of Technological and Biological Approaches to Resilience

Referring to Fig. 1, biological systems commonly use massive load-spreading approaches, i.e., very large numbers of almost identical cells work together for similar functionality within an organ. This approach means that the failure of a single sub-system (cell) has negligible effect on the system (organ). Biological systems almost never use design redundancy (that is, including a workable system that only operates if the main ‘system’ fails).

Figure 1 shows that, in biological systems, resilience is actually provided in distinct layers, this observation might be linked to the concept of survivability - if a failed cell can be replicated before an organ fails, then the functionality of the organ is retained. While many technological systems do illustrate resilience in more than one tier, many examples also illustrate cases where no such layering is seen, and commercial imperatives severely discourage standardization of resilience layers.

Within biological systems, the primary source of information (DNA, with its sequence of A, T, C and G nucleotides) not only exists within each cell, but is replicated when each cell divides and is accessible to the cell’s components directly (i.e., without mediation). Biological systems also contain cellular-level agency that allows information flows from DNA to RNA to proteins - noting that, in biological systems the capability to “read” the information (DNA) that is stored in each cell, is available within that cell. Restated, not only is information stored at cell level, the agency to read the information and carry out the basic processes of replication is present at cellular level.

In many technological system cases, the capacity to replicate a failed subsystem involves diagnosis, proposal, execution, material, and knowledge unavailable at other than international level. Without a nation-state/international level of effort therefore, the subsystem failure could not be fixed and the system would have remained failed. The necessary requirements for resilience can, therefore, be compared by considering the scale at which all of these essential (material, knowledge, and agency) inputs can be made available. For biological systems, highly localised knowledge-base (DNA) and highly localised manipulation (expression) make resilience (replacement of damage) possible via a general-purpose resource (blood supply) that is available to all cells. Similarly, the nutrients and other requirements are themselves generated locally (plant and animal sources) from equally basic and ubiquitous resources. By contrast, replacement of a local component of a technological system may effectively require a financial, transport, offshore raw materials, offshore manufacturing, offshore knowledge-base, and skilled labour resource - and hence only be practical at an international scale. This is an essential difference between the resilience capabilities of biological and current technological systems, and emphasizes that a direction-change is needed to avoid loss of resilience in technological systems and for technological systems to achieve the benchmarks of resilience that are commonly achieved in biological systems.

We also note that resilience within technological systems is currently almost completely dependent upon human capabilities for both abstract problem-solving, and general purpose ‘agency’ – and that the practicality of dependence upon human capabilities to enable resilience is likely to decrease if more integrated systems without layered resilience are implemented.

### The Practical Possibility of Resilient Technological Systems

Considering the practicality of resilience in technological systems, two questions must be answered:

- Are mechanisms used in biological systems inherently incapable of achieving the scope and scale needed to achieve resilience in technological systems, and
- Is it reasonable to foresee capabilities that will allow resilience of technological systems, similar to that displayed by biological systems?

We might ask whether there are any features of technological systems that are sufficiently and intrinsically different from biological systems, that conclusions from biological systems are inapplicable to technological systems. While the scale of some technological systems (undersea cable, 20-storey building, Saturn-5 rocket) are larger than current biological systems, it is difficult to identify a specific reason to believe that this is an inherent limitation although it also invites the counterfactual observation that smaller systems would facilitate resilience. The manipulation of single iron (Fe) ions to become essential to either photosynthesis or oxygen transport by blood show that biological systems are able to manipulate at molecular scale. The formation of bone and tooth enamel show capability for macro-scale manipulation of hard materials: and the development of retinal detectors (rod and cone) show capability to manipulate combinations of photons and electrical/chemical signals. The human brain’s functional capabilities are well-documented. This question can be answered in the negative!

Advances in both small-scale additive and subtractive manufacturing could bring increasingly sophisticated manufacturing capabilities within reach of villages or even individuals, options for small-scale chemical synthesis are at a similar level and availability of knowledge that is both accessible and in a form that is usable (appears to be a reasonable hope). The definitional scope of Artificial General Intelligence (AGI) could include planning the replacement of a failed subsystem, and a synthesis of AGI with a general purpose synthesis facility would indeed duplicate some of the resilience capabilities that are displayed by even the most basic biological system. Within an even shorter timeframe, standardised information formats and general purpose synthesis facilities (possibly with assistance from AI) could see the capability to replace a failed subsystem reduced from international-scale to individual-scale, representing a very significant improvement in resilience!

We conclude that there are no characteristics of technological systems that are sufficiently and intrinsically different from biological systems as to definitely preclude development of resilience approaching that of biological systems. We have however noted (earlier) that trends in technological system development have strongly diverged from the principles that generate resilience in biological systems.

### The Need for Resilient Technological Systems

This paper has specifically considered technological (and extended to socio-technological) issues and avoided either psychological or financial interpretations of “resilience”. Within the technological (and socio-technological) fields, this paper has also intentionally avoided any attempt to exhaustively list technological vulnerabilities or to identify narrowly-focused solutions.

The demonstrated capability of biological systems to maintain functionality over many millennia has been noted and contrasted with technological systems’ typical lifetime (measured in decades): this critical benchmark is that to which technological systems must be measured.

Humanity is increasingly dependent upon technological systems to supply our essential needs and this paper has proposed that there are immediate and significant reasons to believe that current approaches will progressively lose capacity to provide technological systems with essential resilience.

There seems to be an obvious need to review the mechanisms and principles that have enabled biological systems’ demonstrated resilience, and to consider how these might be applied to technological systems now and in the future.

The previous concluding subsections have noted, in principle and with examples, the tendency of technological systems to lose the capability for low-level resilience that exemplify biological systems. Those sections have also examined in principle and with examples both the practicality and the desirability of designing technological systems using principles that are common in biological systems.

We conclude that a) there is real concern, b) there are realistic options for change, and c) there is value in the abstract consideration of “resilience” applied to technological systems.

### Resilient Technological Systems

The evidence that lessons from biological systems are optimal, lies simply in the evidence of their resilience.

In has been proposed that there is no underlying principle that precludes resilience in technological systems (though the timeframe for local scale integrating AGI and full suites of manufacture, is long), and also that there are (strong) pressures upon technological systems, to diverge from the principles that encourage resilience in biological systems.

The characteristic benchmarks that allow resilience in biological systems have been identified and examined, and the translation of these to technological systems can therefore be synthesized in terms of avoidance of issues that preclude resilience (avoiding single points of failure), standardization of components/materials, accessibility of workable knowledge and development of basic manufacture/repair capability, all at very basic level that allows maintenance of high-level functionality during regeneration of failed local and higher-level subsystems.

Such a “recipe” would allow the level of benchmark resilience seen in biological systems to at least be approached by major technological systems, but it is simplistic. Arguably the most important issue is that strong (commercial) trends and pressures are causing technological systems to veer progressively further from the benchmark concepts that have offered biological systems their proven resilience. The development of motivation to adopt principles seen in biological systems, is a large topic and a detailed examination of such motivation is outside the scope of this paper. It is possible that such motivation may be contributed by increased awareness of vulnerability, and assisted by pressures to standardize systems/components. Similarly, the development of technological system case-studies, and the detailed application of the principles identified within biological systems to those case studies, is outside the proposed scope of this paper.

### Beyond Resilience

This analysis has constrained its scope to recovery of functionality following subsystem failure. Supersystem capabilities (abstract and physical) that are shown to be necessary for the recovery of functionality may, with minimal extension, actually enable improved functionality as envisaged in the concept of “anti-fragile” (Taleb, 2013).

## Data Availability

No datasets were generated or analysed during the current study.
